# Chemopreventive Effects of Piper betle (Sirih) on High-Fat Diet-Induced and Azoxymethane-Induced Colon Cancer in Male Sprague-Dawley Rats

**DOI:** 10.7759/cureus.76260

**Published:** 2024-12-23

**Authors:** Nur Hazirah Roslan, Khairul Anwar Zarkasi, Yasmin Anum Mohd Yusof, Suzana Makpol

**Affiliations:** 1 Department of Biochemistry, Faculty of Medicine, Universiti Kebangsaan Malaysia, Kuala Lumpur, MYS; 2 Biochemistry Unit, Preclinical Department, Faculty of Medicine and Defence Health, Universiti Pertahanan Nasional Malaysia, Kuala Lumpur, MYS

**Keywords:** aberrant crypt foci, azoxymethane, chemoprevention, colon cancer, natural product, piper betle

## Abstract

A high-fat diet could lead to obesity, increasing colorectal cancer risk due to dyslipidemia and chronic inflammation, while Piper betle (PB) exhibits anti-tumor, anti-inflammation, and anti-oxidant benefits. This study aimed to determine whether PB possesses chemopreventive effects on high-fat diet (HFD)-induced and azoxymethane (AOM)-induced colon cancer. Male Sprague-Dawley rats receiving either a normal diet or HFD were divided into control, PB, AOM, and AOM+PB subgroups which were then sacrificed after 24 weeks. The lipid profile, leptin, and inflammatory markers were measured from serum, and aberrant crypt foci (ACF) in the colon were detected by methylene blue staining. Cellular proliferation was identified through immunohistochemical staining of antigen Kiel 67 (Ki67) and beta-catenin. There were significant differences in serum total cholesterol, low-density lipoprotein, triglycerides, and high-density lipoprotein in the HFD compared to the normal diet group. The AOM group for normal diet and HFD exhibited significantly increased serum leptin, interleukin-6, IL-12p70, tumor necrosis factor-α, and nuclear factor-κB, with overexpression of Ki67 and beta-catenin. These changes were reversed by PB supplementation. In conclusion, PB demonstrated lipid-modifying and chemopreventive effects against HFD and AOM-induced colon cancer in rats.

## Introduction

According to the Global Cancer Observatory (GLOBOCAN) 2020, various cancer types are responsible for approximately 10 million deaths, globally [1]. The top five common causes of cancer deaths involve the lungs (18%), liver (8.3%), stomach (7.7%), breasts (6.9%), and colorectum (5.8%). In Malaysia, breast cancer ranked first while colorectal cancer (CRC) ranked second among the top-most common cancers according to the National Cancer Registry 2012-2016 [2].

Obesity in the last few decades has been rising in the world population and has been associated with metabolic-related disorders such as diabetes, hypertension, cardiovascular diseases, and certain cancers including esophageal adenocarcinoma, CRC, cholangiocarcinoma, and pancreatic and breast cancers [3]. Systematic reviews and meta-analyses also support the strong association between obesity and CRC [4]. Additionally, the development of obesity is intimately correlated with high-fat diet (HFD) consumption. Recent evidence from a multicenter study has shown that CRC is positively associated with high intake of dietary total fat and cholesterol [5]. Therefore, understanding the correlation between body weight, including HFD consumption, and the risk of CRC is crucial to the understanding of the pathogenesis of CRC-linked obesity and how it can be treated either by weight management, diet modification, or chemopreventive strategies.

Obesity could induce abnormal lipid metabolism and chronic inflammation, resulting in the disruption of adipokines and other hormones. These might play important roles in the search for the underlying molecular mechanism linking obesity and tumorigenesis of colon cancer. In cancer metabolism, an emerging concept explains that the anabolic growth of cancer cells is supported by adipocytes surrounding tumors. These adipocytes provide energy and nutrients by transferring energy-rich metabolites such as fatty acids, ketones, and glutamine [6]. Adipocytes secrete adipokines and several hormones such as leptin [7], inflammatory cytokines (e.g., tumor necrosis factor-alpha, TNF-α) [8], nuclear factor κB (NF-κB) [9], and the interleukin (IL) families (e.g., IL-6) [10].

Piper betle (PB) is a medicinal plant that belongs to the family of Piperaceae, popularly known as a masticating herb in the Southeast Asia region. Research in the past has shown that the leaves of PB possess anti-oxidant activity [11], anti-microbial properties [12], wound-healing properties [13], anti-inflammatory properties [14], and anti-cancer effects [15]. PB is rich in polyphenols that possess anti-oxidant and anti-carcinogenic activities such as betel-phenol, chavibetol, chavicol, and hydroxychavicol [16].

Our previous work reported that PB has anti-oxidant, anti-inflammatory, and anti-tumor effects in *in vitro* and *in vivo* studies. PB induced phase I oxidoreduction, phase II detoxification, and anti-oxidation gene expression by activating the nuclear factor erythroid 2-antioxidant response element (Nrf2-ARE) signaling pathway [17], as well as modulation of anti-oxidant enzymes in aging rats [11]. When combined with ginger extract, PB inhibited synergistically the growth of HCT116 colon cancer cells by inducing caspases-mediated apoptosis [18]. Meanwhile, in azoxymethane (AOM)-induced colon cancer rats, PB exhibited chemopreventive activity via beta-catenin downregulation and KRAS upregulation in the AOM group compared to controls [19].

In the present study, we explored the potential effects of PB as a chemopreventive agent on HFD and AOM-induced colon cancer. This research focused on its impact on (1) lipid parameters, including total cholesterol, triglycerides (TG), high-density lipoproteins (HDL), and low-density lipoproteins (LDL); (2) the formation of aberrant crypt foci (ACF) in the colon; (3) the expression of CRC tumor markers, specifically beta-catenin and Ki67; (4) the levels of pro-inflammatory cytokines such as leptin, IL-6, IL-12p70, and TNF-α; and (4) the activation of the NF-κB inflammatory pathway, which are frequently observed in CRC rat models.

## Materials and methods

Animals

This study received ethical clearance from the Universiti Kebangsaan Malaysia (UKM) Animal Ethics Committee (approval no.: FP/BIOK/2015/YASMIN/29-JULY/690-AUG.-2015-DEC.-2018). Seventy-two, three-week-old male Sprague-Dawley rats were obtained from the Laboratory Animal Resource Unit (LARU), UKM, Kuala Lumpur, Malaysia. The sample size was calculated using the resource equation approach, resulting in a range of 6-11 rats/group. We chose an average of nine rats/group, amounting to 18 rats for two main groups. With four separate analyses conducted under each group, the total number of rats used in the study was 72. The rats were acclimatized for one week and then divided into two major groups which were (i) normal diet (3.8 kcal/g) (*n*=32) and (ii) high-fat diet (HFD) (5.2 kcal/g) (*n*=32). These groups were further divided into four groups each, which include (a) control, (b) PB (150 mg/kg body weight, BW), (c) azoxymethane (AOM), and (d) AOM+PB groups. This specific PB dose was used in this study since previous research has shown that crude PB and purified hydroxychavicol extracts at 150 mg/kg BW exerted anti-ulcer and anticancer activities in animal models [13,15]. The rats were given 24 weeks of either a normal diet or HFD. The standard rodent chow was purchased from Gold Coin Feedmills (Gold Coin Holdings, Singapore). It was stored in a dry place at room temperature and consisted of 3% fat, 21% protein and 5% fibers with a metabolized energy of 2,755 kcal/kg. Meanwhile, HFD was purchased from Altromin (Biosystems Corporation, Singapore). It consisted of 20% fat, 22% protein, and 63% carbohydrate with a metabolized energy of 3,770 kcal/kg. Water was given *ad libitum*. Azoxymethane (AOM) (Sigma, St. Louis, Missouri, USA), a colon cancer-specific carcinogen, was administered intraperitoneally (i.p.) once a week for two consecutive weeks at the dose of 15 mg/kg BW for the AOM and AOM+PB subgroups on the 12thweek of the experiment. Treatment with PB was given once daily from the 12th week onwards for three months via oral gavage to the respective rat groups. All rats were euthanized by decapitation to observe the development of colon tumors and to collect tissues.

Preparation of aqueous extract of PB

The PB leaves obtained from a local supplier were sent for identification and authentication by the Herbarium Department, UKM (voucher no.: UKMB29980). Once collected, the PB leaves were dried under sunlight for a few days and pounded into small pieces. Soxhlet apparatus was used for extraction with a ratio of 1:10 of PB leaves and water for two hours. Thereafter, the PB leaf extract was filtered with filter paper and then centrifuged at 1,000 rpm for 15 minutes. The tubes containing PB leaf extract were kept at -80°C and freeze-dried at -50°C for three days to turn the solution into powder form, which was subsequently stored at 4-8°C until further use. The powder was reconstituted with distilled water at a 1:10 ratio before being administered to rats via oral gavage at the prescribed dose.

Blood and tissue collection

A total of 5 mL of intracardiac blood was withdrawn and placed into a vacutainer tube containing serum-separating gel (Becton, Dickinson & Company, USA). The tube was inverted several times to mix the blood sample with the clot activator. Then, the tube was left at room temperature for 20-30 minutes and then centrifuged at 1,300-1,800 × *g* for 15 minutes. Serum separated from blood cells was collected and stored in a -80°C freezer prior to analysis.

Lipid profile

Measurement of the lipid profile consisting of serum triglycerides (TG), total cholesterol, LDL, and HDL was done by sending the serum for servicing at Quantum Diagnostics (Petaling Jaya, Selangor, Malaysia).

Tumor detection by ACF visualization and quantification

Colons from rats were removed and flushed with normal saline. In addition, the colons from five rats per dietary group were stretched on filter papers and submerged in 10% buffered formalin overnight followed by paraffin embedment. The fixed colon was stained for five minutes with 0.2% methylene blue solution. The total count of ACF was determined in every 2 cm section from the distal to the proximal end of the entire colon for tumor enumeration. Incidence and multiplicity were the parameters used for aberrant crypt assessments. The number of crypts in each focus determined aberrant crypt multiplicity and categorized as the presence of ≥4 aberrant crypts/focus.

Immunohistochemistry for the detection of Ki67 and beta-catenin

Colons from sacrificed rats were fixed in formalin overnight and embedded in paraffin. Proliferation was evaluated through antigen Kiel 67 (Ki67) and beta-catenin using an Abcam Kit (Cambridge, England) according to the manufacturer’s instructions. Briefly, paraffin-embedded tissues were cut at 4 μm thickness and mounted on slides coated with poly-L-lysine. Archival samples were dewaxed by gradually washing them in xylene and alcohol. Slides were then incubated with 3% hydrogen peroxide to quench the activity of endogenous peroxide. The colon tissues were then incubated with primary antibody for Ki67 (ab21700) at 1:50 dilution and beta-catenin (ab630) at 1:1000 for 35 minutes. The labeled streptavidin-biotin 2 (LSAB2) system-horse radish peroxidase (HRP) (Dako, Glostrup, Denmark) was used as a secondary antibody. The peroxidase activity was visualized by incubating with diaminobenzidine for five minutes followed by counterstaining with hematoxylin for 15 seconds. The positively stained cells were observed under a light microscope and evaluated with a semiquantitative scoring system as follows: three for strongly stained (>50% cells stained); two for moderately stained (31-50% cells stained); one for weakly stained (11-30% cells stained); and zero for negatively stained (<11% cells stained).

Determination of serum leptin and inflammatory markers

Serum samples from all rats were analyzed for leptin and inflammatory markers including IL-6, IL-12p70, TNF-α, and NF-κB using the respective enzyme-linked immunosorbent assay (ELISA) kits. Serum leptin, IL-6, IL-12p70, and TNF-α were measured using the Milliplex Map Rat Serum Adipokine Panel (Millipore, Massachusetts, USA). The serum NF-κB was measured using the Rat NF-κB-p65 ELISA Kit (Elabscience, China). Briefly, serum samples of rats obtained from all dietary groups were reacted with reagents in the respective kits and analyzed spectrophotometrically at 450 nm using a microplate reader. By referring to the kits’ standards, serum levels of leptin, IL-6, IL-12p70, TNF-α, and NF-κB were calculated and expressed as pg/mL.

Statistical analysis

The data of ACF were expressed as mean ± standard error of the mean (SEM), and the differences were assessed using one-way analysis of variance (ANOVA). The scoring of Ki67 and beta-catenin protein biomarker expression was based on the H-score, and the Kruskal-Wallis H test was applied to assess the statistical differences. Other parameters were reported as mean ± standard deviation (SD) and then analyzed by one-way ANOVA. Significant differences were determined when *p*<0.05. All statistical analyses were performed using IBM SPSS Statistics for Windows, Version 20 (Released 2011; IBM Corp., Armonk, New York, United States).

## Results

Body weight

**Figure 1:**
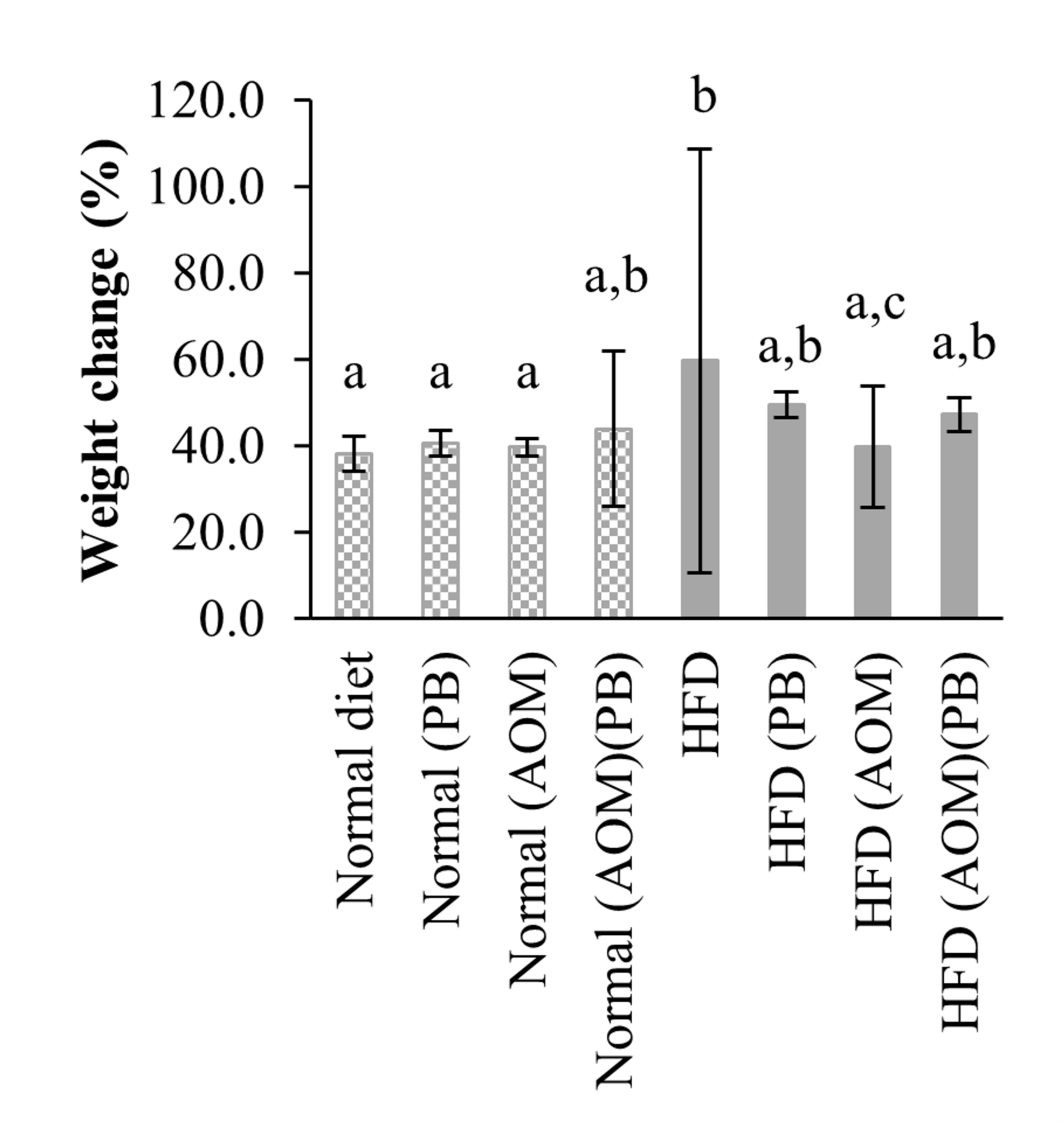
Mean weight changes over the course of experiment. Groups marked with different letters were statistically different at *p*<0.05. AOM: azoxymethane; HFD: high-fat diet; PB: Piper betle.

The rats receiving HFD gained significantly higher amounts of weight compared to rats receiving a normal diet (+59.7% *vs* +38.2%, *p*<0.05) (Figure 1). Induction of colon cancer by AOM in HFD rats significantly reduced their weight compared to the HFD group (+39.9% *vs* +59.7%, *p*<0.05). Otherwise, the weight gains among the remaining rat groups were statistically equal.

Lipid profile

**Figure 2:**
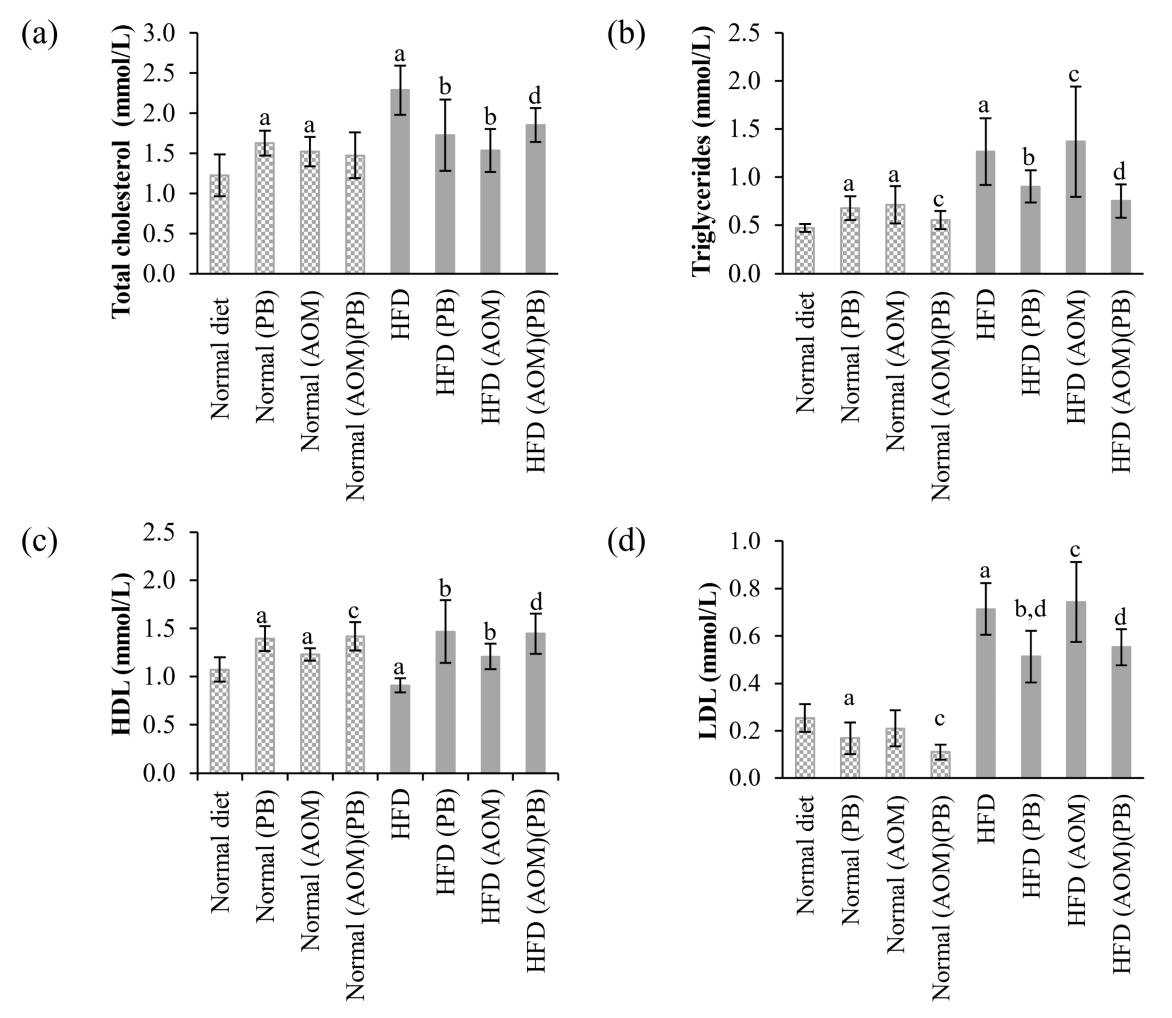
Effects of high-fat diet (HFD) and PB supplementation on the lipid profile of (a) total cholesterol, (b) triglyceride, (c) HDL, and (d) LDL in each dietary group. Small letters on the bar graph denote significant differences at *p*<0.05 compared to ^a^Normal diet, ^b^HFD, ^c^Normal (AOM), and ^d^HFD (AOM) groups. AOM: azoxymethane; HDL: high-density lipoprotein; HFD: high-fat diet; LDL: low-density lipoprotein; PB: Piper betle.

There were significant differences in lipid profiles among all dietary groups. Total cholesterol increased significantly (*p*<0.05) in the HFD group when compared with the normal diet group (Figure 2a). Supplementation with PB reduced total cholesterol levels in the HFD group but not in the normal diet group. Triglycerides (TG) and LDL showed similar patterns, wherein the HFD group, either with AOM or without AOM, had significantly increased TG and LDL levels (*p*<0.05) compared to the normal diet group. Treatment with PB reduced TG and LDL for all dietary groups (Figures 2b, 2d). The HDL level was reduced significantly in the HFD group, and interestingly, treatment with PB increased its level across all dietary groups (Figure 2c).

ACF formation

**Figure 3:**
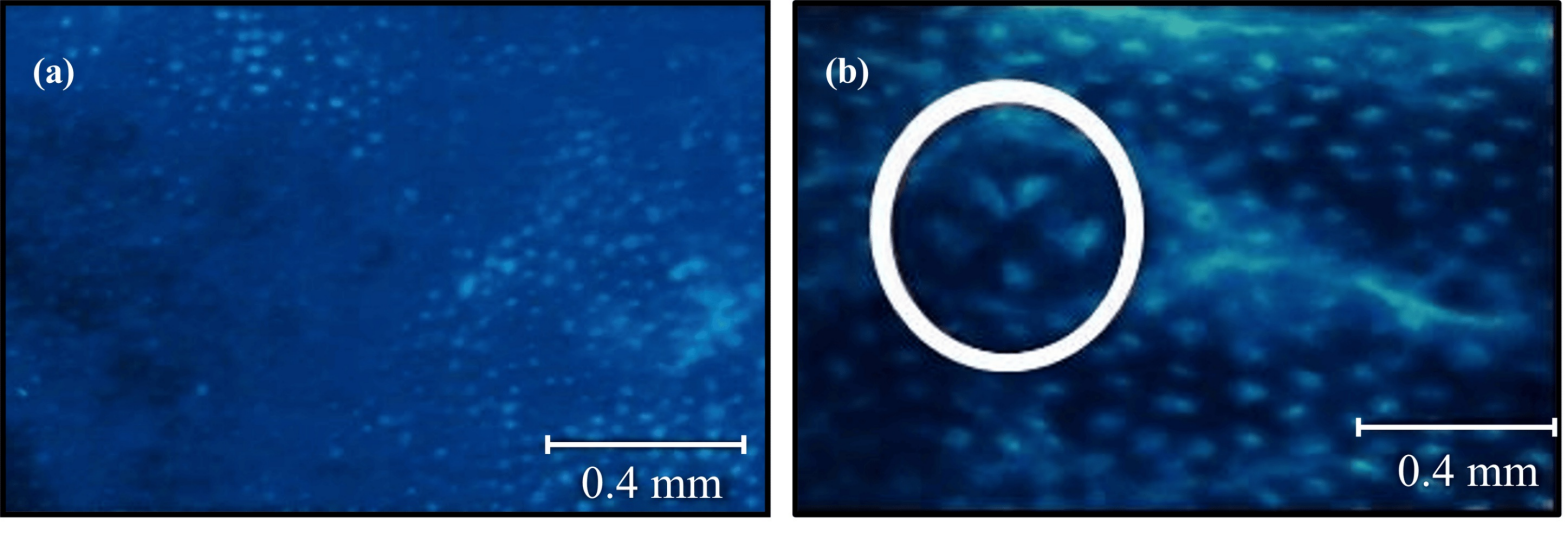
Methylene blue staining of (a) normal colon and (b) AOM-induced colon cancer at 40× magnification. AOM: azoxymethane.

**Figure 4:**
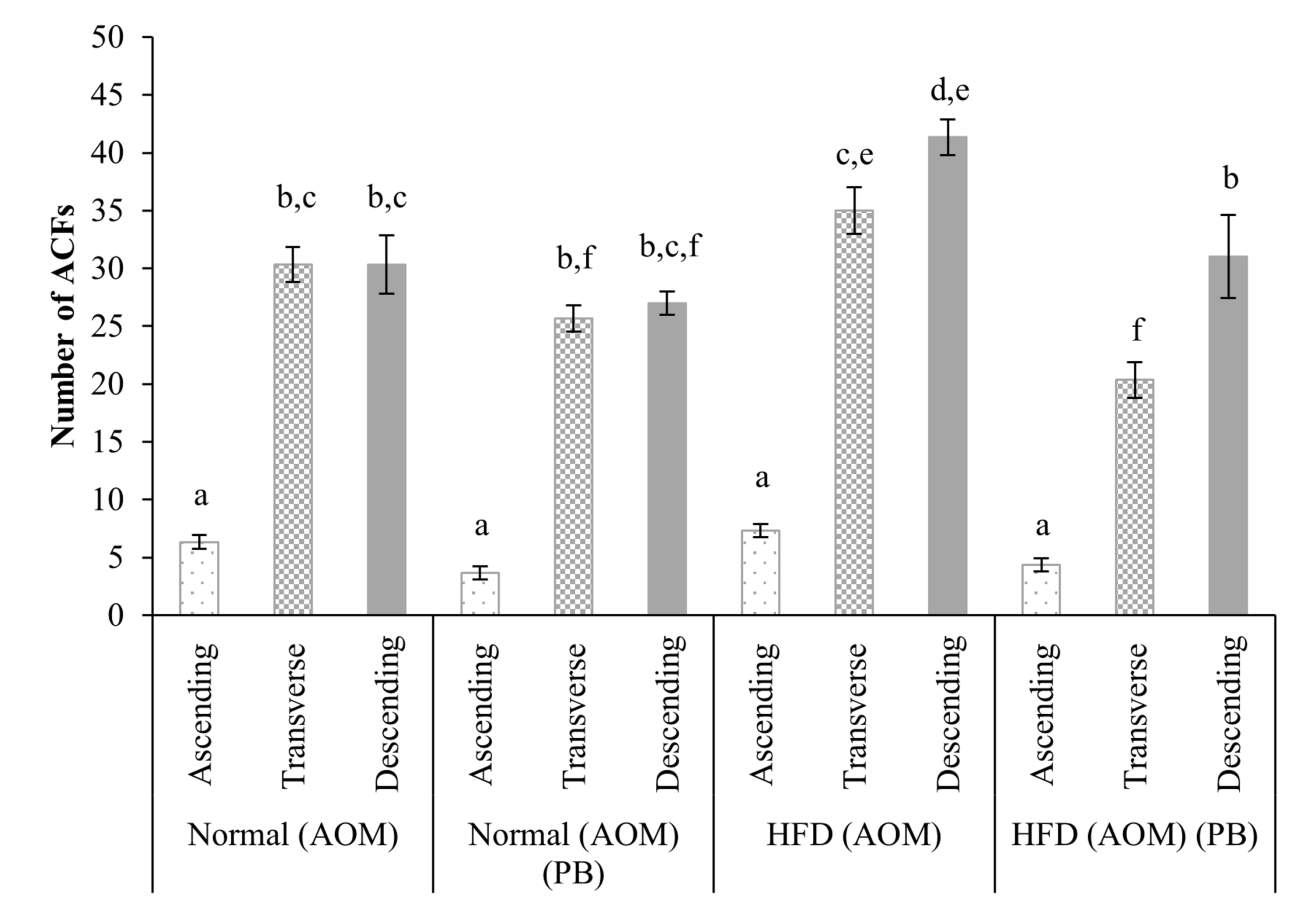
Number of aberrant crypt foci (ACF) in HFD-induced colon cancer (AOM) and PB-treated groups at the ascending, transverse, and descending colons. Groups marked with different letters were statistically different at *p*<0.05. Data are presented as mean ± SEM for five samples from each group. ACF: aberrant crypt foci; AOM: azoxymethane; HFD: high-fat diet; PB: Piper betle*;* SEM: standard error of mean.

The methylene blue staining of control rats displayed a normal crypt size while AOM-induced colon cancer rats displayed the presence of ACF (Figures 3a, 3b). To examine the impact of HFD on AOM-induced colon cancer and to determine whether HFD might act as a tumor promoter, we investigated the formation of ACF by chemical induction as an indicator of colon carcinogenesis. The most abundant part of the colon with ACF was in the transverse colon, followed by the descending and the ascending colon. HFD fed to AOM rats produced significantly more ACF than AOM rats alone as observed in the transverse colon and descending colon. Treatment with PB significantly reduced the formation of ACF in both AOM and HFD+AOM groups (Table 1, Figure 4).

**Table 1:** Number of ACF in HFD and PB rats induced with colon cancer (AOM). ACF: aberrant crypt foci; AOM: azoxymethane; HFD: high-fat diet; PB: Piper betle.

Group	Ascending colon (*n*)	Transverse colon (*n*)	Descending colon (*n*)
AOM	6	30	30
AOM+PB	4	26	27
AOM+HFD	7	35	41
AOM+HFD+PB	4	20	31

Assessment of tumor cell proliferation by immunohistochemical staining

Ki67

**Figure 5:**
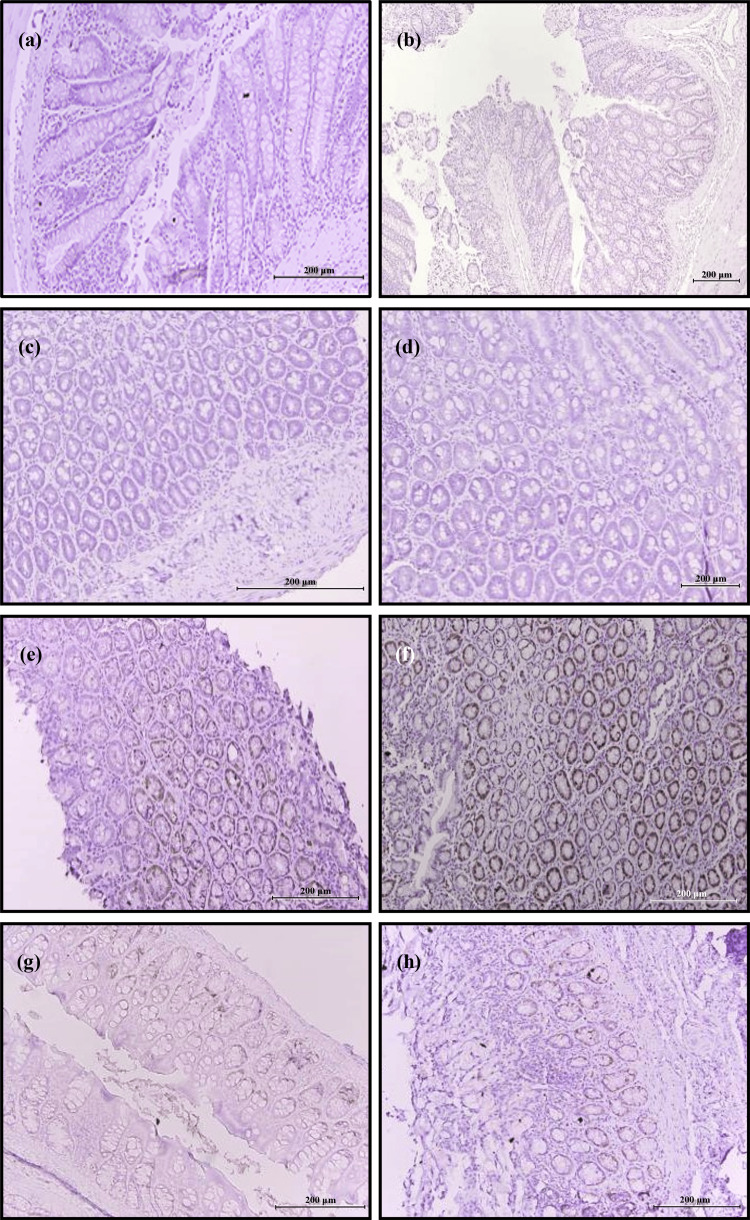
Effects of PB on the expression of Ki67 by colon tissues in various dietary groups examined under a light microscope at 200× and 400× magnifications. (a) Normal diet, (b) HFD, (c) Normal diet+PB, (d) HFD+PB, (e) Normal diet+AOM, (f) HFD+AOM, (g) Normal diet+AOM+PB, and (h) HFD+AOM+PB. AOM: azoxymethane; HFD: high-fat diet; Ki67: antigen Kiel 67; PB: Piper betle.

Observation under the light microscope showed that cell proliferation determined by Ki67 expression was minimal in normal diet, HFD, normal diet+PB, and HFD+PB groups (Figures 5a-5d). A substantial increase in cell proliferation was seen in normal diet+AOM and HFD+AOM groups, with the latter having maximal Ki67 expression (Figures 5e, 5f). Supplementation with PB to rats in both normal diet+AOM+PB and HFD+AOM+PB groups resulted in a marked reduction of Ki67 expression (Figures 5g, 5h).

**Figure 6:**
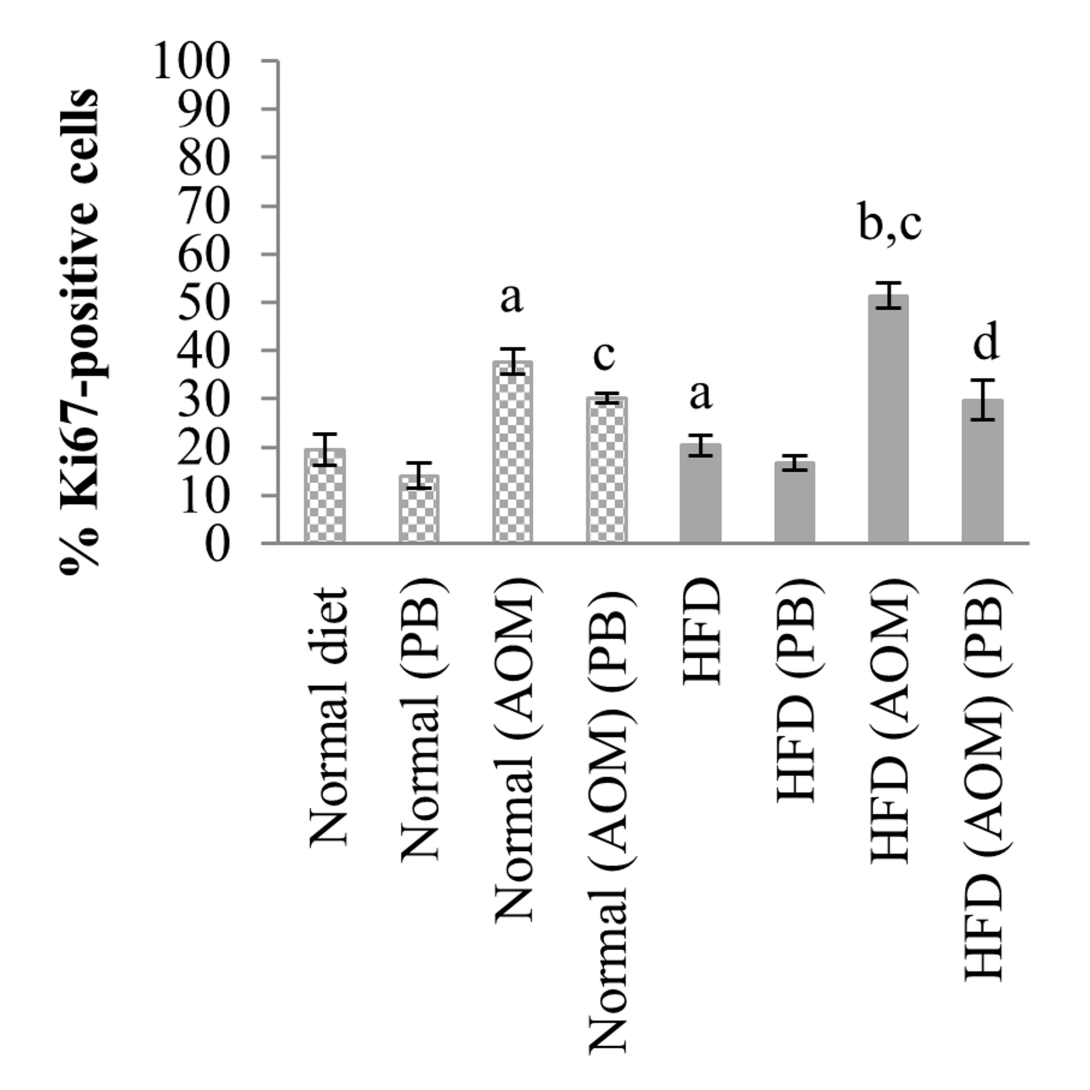
Effects of PB on the percentages of Ki67-positive cells of colon cancer-induced rats. Small letters on the bar graph denote significant differences at *p*<0.05 compared to ^a^Normal diet, ^b^HFD, ^c^Normal (AOM), and ^d^HFD (AOM) groups. AOM: azoxymethane; HFD: high-fat diet; Ki67: antigen Kiel 67; PB: Piper betle.

Quantitative analysis revealed that rats receiving HFD had a slight but significant increase in Ki67 expression compared to the normal diet group (20% *vs* 19%, *p*<0.05) (Figure 6). Colon cancer induced by AOM increased Ki67-positive cells by 19% compared to normal diet control (38% *vs* 19%, *p*<0.05), whereas HFD+AOM-induced colon cancer showed 31% increased Ki67-positive cells compared to the HFD group (50% *vs* 20%, *p*<0.05). Treatment with PB significantly inhibited cell proliferation in both AOM and HFD+AOM-induced colon cancer rats. The reduction was by 8% in normal diet+AOM+PB compared to normal diet+AOM and 21% in HFD+AOM+PB compared to HFD+PB groups (both *p*<0.05).

Beta-Catenin

**Figure 7:**
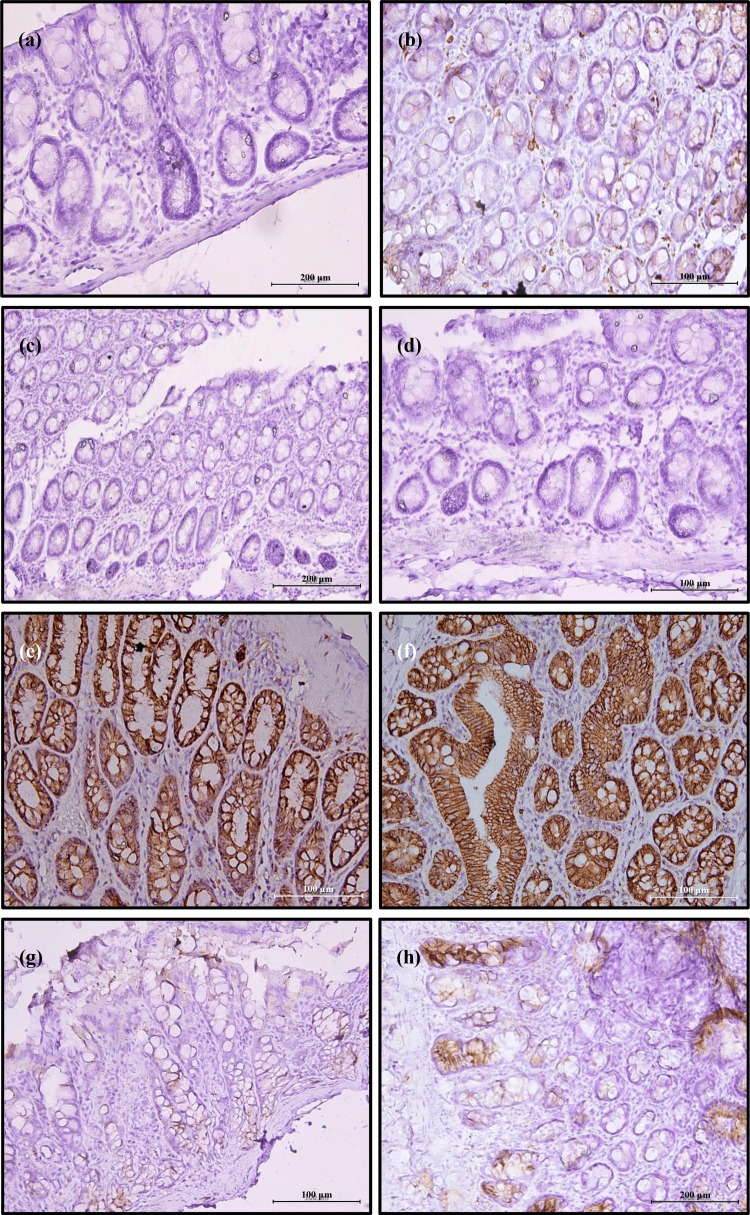
Effects of PB on the expression of beta-catenin by colon tissues in various dietary groups examined under a light microscope at 200× and 400× magnifications. (a) Normal diet, (b) HFD, (c) Normal diet+PB, (d) HFD+PB, (e) Normal diet+AOM, (f) HFD+AOM, (g) Normal diet+AOM+PB, and (h) HFD+AOM+PB. AOM: azoxymethane; HFD: high-fat diet; PB: Piper betle.

Similar patterns with Ki67 could be seen for the immunoreactivity of beta-catenin, in which, minimal expression was observed in the normal diet, HFD, normal diet+PB, and HFD+PB groups (Figures 7a-7d). The expression of beta-catenin was the highest in HFD+AOM (Figure 7f) followed by normal diet+AOM groups (Figure 7e). Marked reduction of beta-catenin expression occurred as a result of PB supplementation in normal diet+AOM+PB and HFD+AOM+PB rat groups (Figures 7g, 7h).

**Figure 8:**
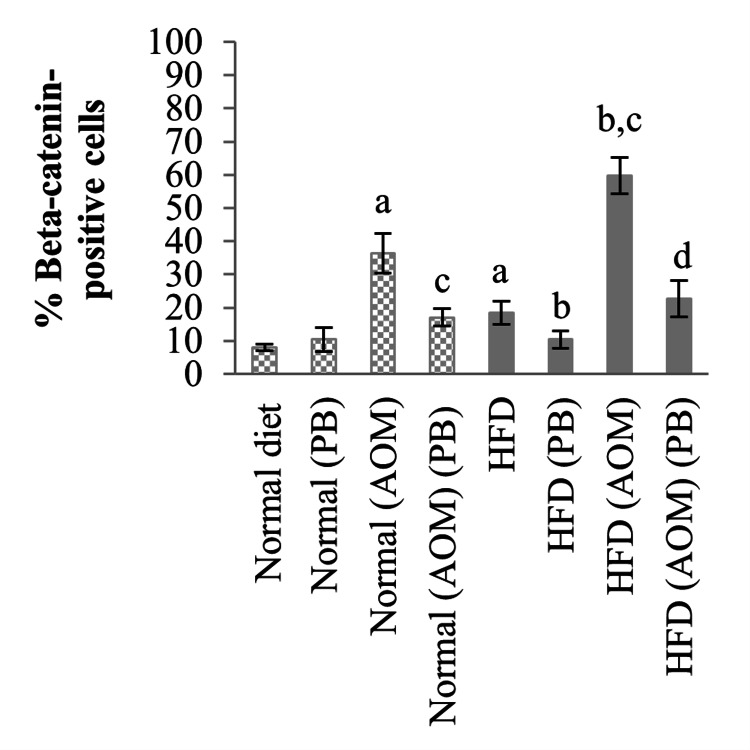
Effects of PB on the percentages of beta-catenin positive cells of colon cancer-induced rats. Small letters on the bar graph denote significant differences at *p*<0.05 compared to ^a^Normal diet, ^b^HFD, ^c^Normal (AOM), and ^d^HFD (AOM) groups. AOM: azoxymethane, HFD: high-fat diet, PB: Piper betle.

The percentage of beta-catenin-positive cells in rats fed with HFD increased by 10% compared to the normal diet group (18% *vs* 8%, *p*<0.05) (Figure 8). Administration of AOM increased beta-catenin expression by 28% in the normal diet+AOM group compared to normal diet control (36% *vs* 8%, *p*<0.05) and 42% in the HFD+AOM group compared to HFD rats (60% *vs* 18%, *p*<0.05). Like Ki67 expression, PB supplementation remarkably reduced beta-catenin expression by 19% in the normal diet+AOM+PB compared to the normal diet+AOM group and 37% in HFD+AOM+PB than HFD+AOM group (both *p*<0.05).

Evaluation of leptin and inflammatory markers

Serum Leptin

**Figure 9:**
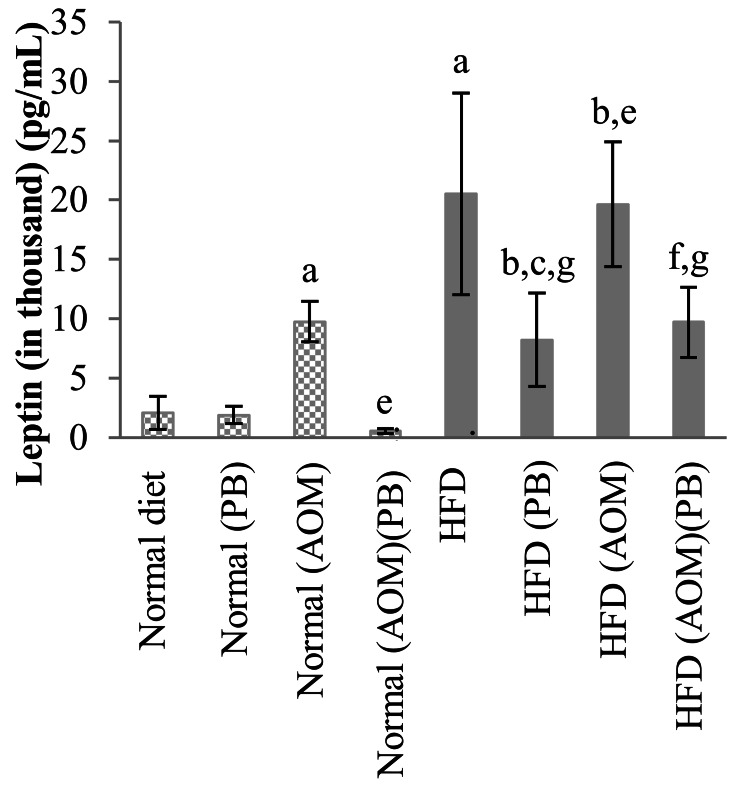
Effects of PB on serum leptin levels. Small letters on the bar graph denote significant differences at *p*<0.05 compared to ^a^Normal diet, ^b^HFD, ^c^Normal (PB), ^d^HFD (PB), ^e^Normal (AOM), ^f^HFD (AOM), and ^g^Normal (AOM) (PB) groups. AOM: azoxymethane; HFD: high-fat diet; PB: Piper betle.

Serum levels of leptin hormone and inflammatory markers comprising IL-6, IL-12p70, TNF-α, and NF-κB were evaluated by ELISA. Leptin increased significantly in HFD-fed rats than normal diet control (Figure 9). Administration of AOM caused elevation of leptin in normal diet+AOM compared to normal diet control, as well as in HFD+AOM compared to the HFD group. These changes were significantly reversed by PB supplementation which were apparent in normal diet+AOM+PB compared to normal diet+AOM and HFD+AOM+PB compared to HFD+AOM (all *p*<0.05).

Serum IL-6, IL-12p70, and TNF-α

**Figure 10:**
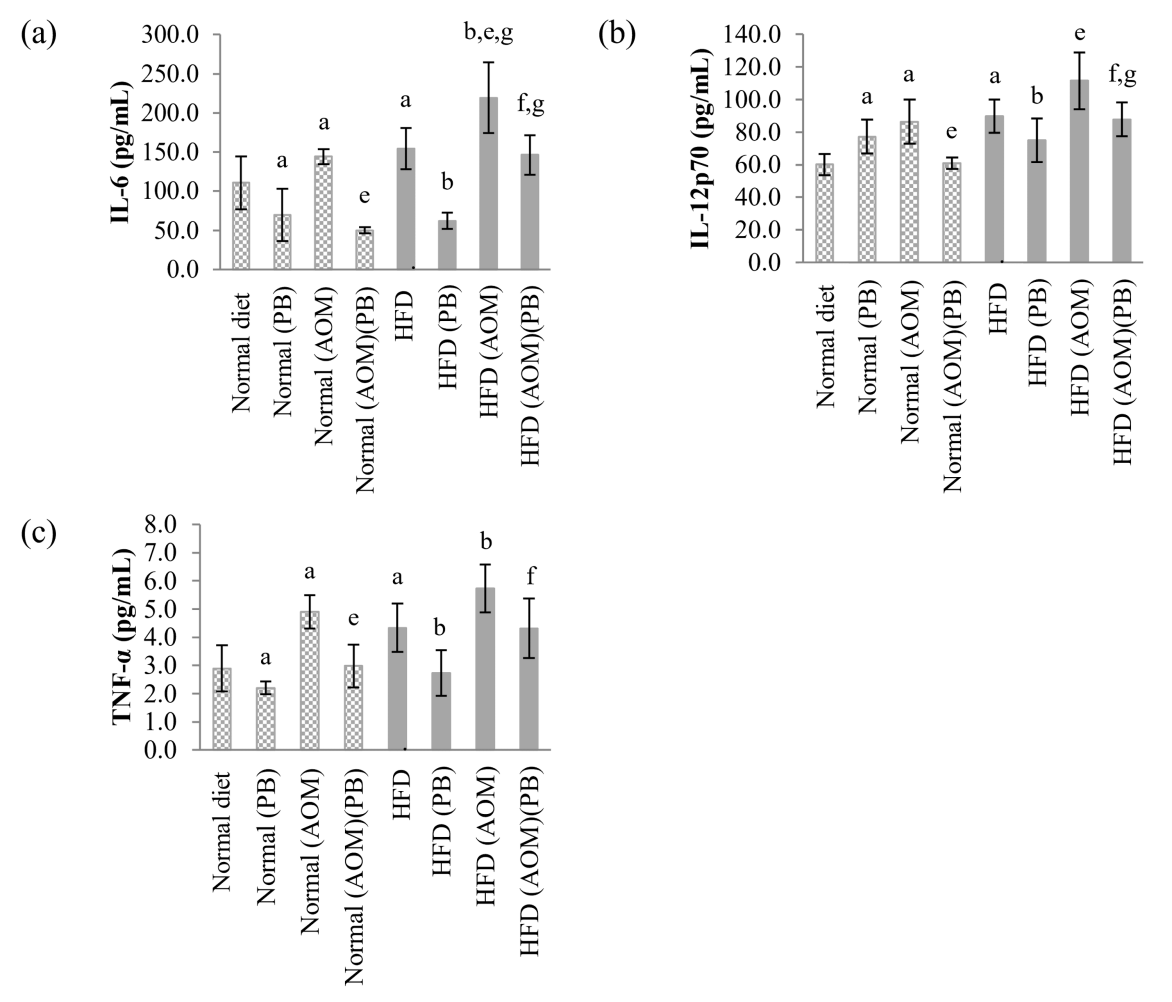
Effects of PB on serum (a) IL-6, (b) IL-12p70, and (c) TNF-α levels. Small letters on the bar graph denote significant differences at *p*<0.05 compared to ^a^Normal diet, ^b^HFD, ^c^Normal (PB), ^d^HFD (PB), ^e^Normal (AOM), ^f^HFD (AOM), and ^g^Normal (AOM) (PB) groups. AOM: azoxymethane; HFD: high-fat diet; IL: interleukin; PB: Piper betle*;* TNF: tumor necrosis factor.

All three cytokines, the IL-6, IL-12p70, and TNF-α, showed similar changes whereby they were significantly elevated in HFD-fed rats compared to normal diet control (Figure 10). Induction of colon cancer by AOM caused elevation of IL-6, IL-12p70, and TNF-α in normal diet+AOM compared to normal diet control. In contrast, HFD+AOM rats only showed increased serum IL-6 and TNF-α compared to the HFD group but not for IL-12p70. Serum levels for all three cytokines were significantly decreased following PB supplementation in normal diet+AOM+PB and HFD+AOM+PB groups as compared to normal diet+AOM and HFD+AOM, respectively.

Serum NF-κB

**Figure 11:**
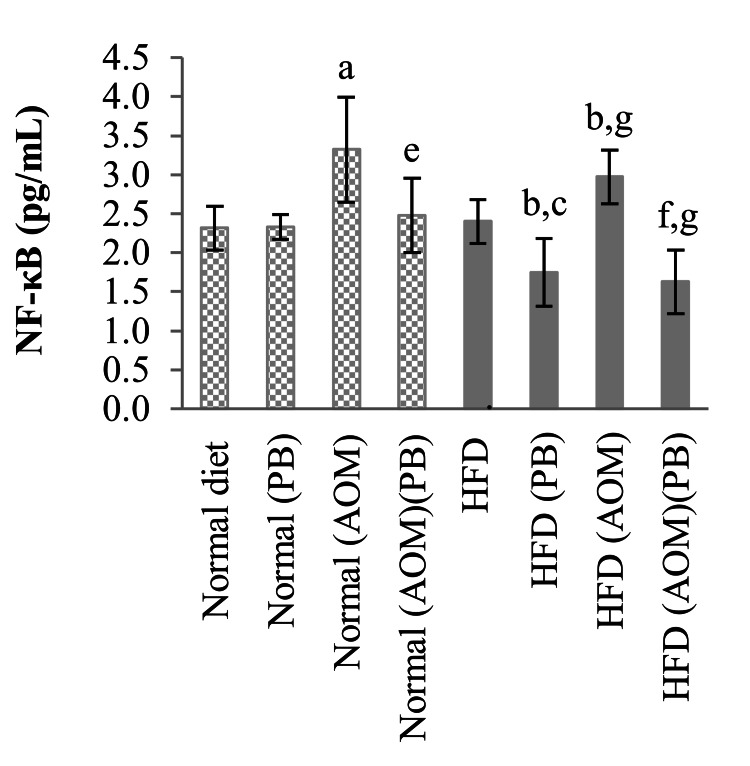
Effects of PB on serum NF-κB levels. Small letters on the bar graph denote significant differences at *p*<0.05 compared to ^a^Normal diet, ^b^HFD, ^c^Normal (PB), ^d^HFD (PB), ^e^Normal (AOM), ^f^HFD (AOM), and ^g^Normal (AOM) (PB) groups. AOM: azoxymethane; HFD: high-fat diet; NF-κB: nuclear factor κB; PB: Piper betle.

There were similar serum NF-κB levels between rats in the HFD and normal diet groups (Figure 11). Induction of colon cancer resulted in significant upregulation of NF-kB in normal diet+AOM compared to normal diet control as well as HFD+AOM compared to the HFD group. These changes were reversed by PB supplementation which were exhibited by normal diet+AOM+PB and HFD+AOM+PB compared to normal diet+AOM and HFD+AOM, respectively.

## Discussion

Obesity is associated as a risk factor in the development of cancers of various organs such as the breast, ovary, prostate, liver, pancreas, and colorectum. Global patterns in excess body weight with high body mass index (BMI) (>30 kg/m^2^) and its association with cancer burden have been studied using meta-analysis [20]. The connection between obesity and the risk of cancer development and recurrence is not fully understood and it may involve fatty acid dysmetabolism, adipokines secretion, chronic inflammation, and dysregulation of cancer signaling pathways [20]. Weight-reducing strategies as well as consuming flavonoids-phenol-rich natural foods known to have chemopreventive properties in targeting phosphatidylinositol 3-kinase/protein kinase B/mammalian target of rapamycin (PI3K/Akt/mTOR) and Wnt-beta-catenin signaling pathways are strategic interventions as a key component of cancer treatment and prevention. Natural therapies with phenol-rich plant products reduce the adverse side effects of cancer treatment seen with chemotherapy drugs [21].

The AOM animal model represents the model of sporadic colon carcinogenesis in humans and shows similarity in terms of clinical symptoms and pathological findings. The model induces the formation of aberrant crypt foci, with crypt multiplicity known to progress to colon cancer in obesity-induced mice [22]. We were able to show the formation of ACF mainly in the middle and distal region of the colon similar to the findings of previous researchers [19,22]. Interestingly when PB was fed to rats, it reduced the formation of ACF significantly showing its anti-tumor effects.

The Wnt-beta-catenin signaling pathway is hyperactivated in almost all CRCs and is believed to be the initiating event in CRC. Dysregulation of the pathway produces undegradable beta-catenin protein that accumulates in the cytosol and nucleus of colon cancer cells. Increased expression of beta-catenin is associated with tumor cell proliferation and stem-like phenotypes [23]. We found an increased expression of beta-catenin in AOM rats as well as in HFD-AOM rats compared to normal dietary groups, while PB was able to downregulate its expression perhaps by inhibiting the Wnt signaling pathway and causing its degradation. The effect may be associated with the flavonoids found in PB such as hydroxychavicol, chavibetol, allylpyrocatechol, and eugenol [24,25]. A similar finding by Al-Henhena et al. (2015) shows the chemopreventive and anti-oxidant effect of *Strobilanthes crispus* leaf extract in reducing the number of ACF as well as the expression of proliferating cell nuclear antigen (PCNA), B-cell lymphoma 2 (Bcl2), and beta-catenin [22].

A meta-analysis study among colorectal cancer patients found that high expression of Ki-67 was significantly associated with poor overall survival. Ki-67 protein expression denotes cell proliferation-associated nuclear protein which is usually expressed in all phases of the cell cycle and its expression is a reliable means of evaluating tumor cell proliferation [26]. The downregulation of Ki-67 expression was also seen in PB-fed colon cancer rats both in AOM alone and in AOM-HFD rats in the current study. The expression of Ki-67 is regulated by the c-Jun N-terminal kinase (JNK) signaling pathway. In a previous study, hydroxychavicol, an active compound purified from PB, has been shown to activate the JNK pathway in HT-29 colon cancer cells [27]. Activation of the JNK pathway resulted in cell cycle arrest at the G_0_/G_1_ phase, resulting in the HT-29 colon cancer cellular apoptosis [27]. Increased cell death correlated with decreased Ki-67 expression, linking the anti-cancer mechanism of PB with the JNK pathway.

Our HFD model exhibited weights similar to the normal diet group at the end of the 24-week experiment. However, the weight gained by the HFD group was significantly higher than the normal diet group, as illustrated by the mean weight changes. This observation is consistent with previous studies, in which, the administration of a high-carbohydrate and high-fat (HCHF) diet did not cause elevated body weight in rats despite causing overt hypertriglyceridemia [28]. Nonetheless, these rats were proven to have developed central obesity by measuring their fat percentages using a dual-energy X-ray absorptiometry (DEXA) scan, later confirmed by *ex vivo* analysis of the omental fat tissues [28]. We hypothesized that rats receiving HFD had a faster rate of adipose accumulation while rats receiving normal diets had a slower rate of lean tissue growth. Lean tissue is denser than adipose, contributing to these rats' statistically similar final weights.

In our study, the lipid profile of HFD-fed rats showed dyslipidemia with significantly increased cholesterol, LDL, and TG and decreased HDL as compared to the normal dietary rats (normal and PB-fed rats). These changes also served as indirect indicators of central obesity, as reported in previous research [28]. When PB was fed to the normal and cancer-induced rats, it significantly reduced cholesterol, LDL, and TG, along with increased HDL, showing its lipid-modifying effects. The latter could be due to the phenolic compounds found in PB such as chavibetol, allylpyrocatechol, and eugenol, as previously mentioned [23,24].

Inflammation associated with obesity is primarily caused by excess nutrients that activate the signaling pathways localized in adipose tissue triggering inflammatory responses resulting in the increased secretion of cytokines such as IL-6, TNF-α, IL-17, and IL-23; c-JNK; and NF-κB [29]. Our results showed that IL-6, IL-12, TNF-α, and NF-κB increased significantly in the AOM group for both normal diet and HFD but decreased significantly when treated with PB showing that PB has anti-inflammatory effects in cancer cells. Leptin and adiponectin dysregulation that occurs in obesity may have a role in obesity-associated colorectal cancer whereby high leptin levels are associated with an increased colon cancer risk [7,30]. In our study, we found that leptin is raised in colon cancer-induced rats and obesity-induced colon cancer rats, while PB was able to suppress these changes.

This study has several limitations. Firstly, it was conducted only at the protein level, targeting selected potential biomarkers and signaling pathways due to financial and time constraints. Therefore, further investigations using transcriptomics and proteomics analyses are necessary to uncover the overall mechanisms and pathways associated with PB chemoprevention in CRC. Secondly, the ideal dosing for PB remains unclear due to the lack of dose-response research. Thirdly, the understanding of PB's bioavailability and metabolism is constrained by the absence of pharmacokinetic data. Consequently, while the current study focused on the chemopreventive effects of PB on HFD and AOM-induced CRC, these limitations need to be addressed in future research. Lastly, the study used an animal model to induce CRC and assess the chemopreventive activities of PB, which may not be directly translatable to humans due to potential interactions between genetics, drugs (e.g., polyphenols in PB), and environmental factors. Thus, investigations in human subjects are necessary to support the clinical use of PB in CRC prevention. Addressing these issues will facilitate the translation from animal studies to clinical use of PB extract specifically among CRC patients, followed by carefully designed Phase I-III clinical trials to ensure the safety and efficacy of PB extract in clinical settings.

Despite these limitations, the current study's novelty was highlighted in several key aspects, including the use of a specific animal model, comprehensive biomarker analysis, and long-term study duration. Previous PB research specifically on colon cancer was performed on HT29 and HCT116 cell lines, as well as in rats with AOM-induced colon cancer. In contrast, the current study utilized a colon cancer rat model induced by the combination of HFD and AOM. This strategy closely mimicked human conditions and provided more relevant information for potential clinical applications. Additionally, the current study measured a wide range of biomarkers, including lipid profile, leptin, and inflammatory markers comprising IL-6, IL-12p70, TNF-α, and NF-κB. This provides a more holistic view of PB's effects, not only on colon cancer but also on general health. Moreover, the study was conducted over 24 weeks, allowing the long-term effects of PB supplementation to be observed.

## Conclusions

The combined effects of HFD and AOM significantly caused hyperlipidemia particularly involving serum triglyceride and LDL levels. Rats exposed to both HFD and AOM developed higher numbers of ACF with increased expressions of Ki67 and beta-catenin. Moreover, HFD and AOM exposure caused increased expression of serum leptin as well as systemic inflammation marked by elevated serum IL-6, IL-12p70, TNF-α, and NF-κB. All of these changes were significantly reversed by PB supplementation. These findings support the anti-inflammatory and anti-tumor effects of PB and serve as preliminary evidence for its potential use as a chemopreventive agent. Nevertheless, further research is needed to clarify PB's lipid-modifying effects and its role in obesity-related colon cancer. While our data support the anti-inflammatory and chemopreventive effects of PB, more comprehensive molecular evidence and dose-dependent studies are required to establish the precise mechanisms of PB to facilitate PB use in clinical translation.
